# Harmonization of multi-site functional MRI data with dual-projection based ICA model

**DOI:** 10.3389/fnins.2023.1225606

**Published:** 2023-07-20

**Authors:** Huashuai Xu, Yuxing Hao, Yunge Zhang, Dongyue Zhou, Tommi Kärkkäinen, Lisa D. Nickerson, Huanjie Li, Fengyu Cong

**Affiliations:** ^1^School of Biomedical Engineering, Dalian University of Technology, Dalian, China; ^2^Faculty of Information Technology, University of Jyväskylä, Jyväskylä, Finland; ^3^McLean Imaging Center, McLean Hospital, Belmont, MA, United States; ^4^Department of Psychiatry, Harvard Medical School, Boston, MA, United States; ^5^School of Artificial Intelligence, Faculty of Electronic Information and Electrical Engineering, Dalian University of Technology, Dalian, China; ^6^Key Laboratory of Integrated Circuit and Biomedical Electronic System, Dalian University of Technology, Dalian, China

**Keywords:** multi-site, site effects, functional magnetic resonance imaging, independent component analysis, dual-projection

## Abstract

Modern neuroimaging studies frequently merge magnetic resonance imaging (MRI) data from multiple sites. A larger and more diverse group of participants can increase the statistical power, enhance the reliability and reproducibility of neuroimaging research, and obtain findings more representative of the general population. However, measurement biases caused by site differences in scanners represent a barrier when pooling data collected from different sites. The existence of site effects can mask biological effects and lead to spurious findings. We recently proposed a powerful denoising strategy that implements dual-projection (DP) theory based on ICA to remove site-related effects from pooled data, demonstrating the method for simulated and *in vivo* structural MRI data. This study investigates the use of our DP-based ICA denoising method for harmonizing functional MRI (fMRI) data collected from the Autism Brain Imaging Data Exchange II. After frequency-domain and regional homogeneity analyses, two modalities, including amplitude of low frequency fluctuation (ALFF) and regional homogeneity (ReHo), were used to validate our method. The results indicate that DP-based ICA denoising method removes unwanted site effects for both two fMRI modalities, with increases in the significance of the associations between non-imaging variables (age, sex, etc.) and fMRI measures. In conclusion, our DP method can be applied to fMRI data in multi-site studies, enabling more accurate and reliable neuroimaging research findings.

## 1. Introduction

Functional magnetic resonance imaging (fMRI) has become a popular tool for understanding the human brain and detecting brain diseases since its inception in the 1990s (Eklund et al., 2016). Over the past three decades, countless methods and paradigms have been adopted to utilize and interpret fMRI data. One popular approach is frequency-domain analysis. Zang et al. proposed the amplitude of low frequency fluctuations (ALFF) for a voxel’s time series, and it measures the total signal power in the low-frequency range is computed as the total power in the low frequency range (0.01–0.1 Hz; Zang et al., 2007). Another popular approach is based on regional homogeneity analysis, or ReHo (Zang et al., 2004), which computes a voxel-based measure of brain activity that evaluates the synchronization between the time series of a given voxel and its nearest neighbors using Kendall’s coefficient of concordance that is used to investigate the local coherence of fMRI signals in the brain (Yang et al., 2020).

Most neuroimaging studies are conducted within a single research site, with limited capabilities for collecting large sample-size datasets. Smaller sample sizes and lack of harmonization across independent studies present challenges in achieving acceptable reliability and reproducibility of neuroimaging research (Nichols et al., 2017). As a result, multi-site fMRI studies are becoming increasingly common to increase the power of statistical analyses to detect group differences, longitudinal changes, and, in turn the reliability and reproducibility of neuroimaging research. While combining multiple datasets across different studies is beneficial for the development of neuroscience, the existence of site effects makes pooling multi-site datasets challenging. Site effects can confound actual effects of interest and make the final results hard to interpret for fMRI data (Biswal et al., 2010; Groves et al., 2011; Li et al., 2020). Hence, effectively eliminating or minimizing the site effect is necessary for the fusion of multi-site fMRI data.

Recently, a new technique, independent component analysis (ICA) with dual-projection (ICA-DP), was proposed for removal of site effects in multi-site structural MRI data (Hao et al., 2023). For ICA-DP, mixed components are separated into a part related to signal only and a part related only to noise by applying a projection procedure. The noise effects extracted from the mixed components *via* the projection step and other pure noise components are then removed from the data using a second projection procedure. Compared with traditional ICA and ComBat (COMbining BATches), which is a general linear modal (GLM)-derived method based on the empirical Bayes approach (Johnson et al., 2007; Stein et al., 2015; Fortin et al., 2017, 2018; Beer et al., 2020; Cetin-Karayumak et al., 2020; Da-ano et al., 2020; Pinto et al., 2020; Cackowski et al., 2021; Eshaghzadeh Torbati et al., 2021; Maikusa et al., 2021; Bell et al., 2022; Orlhac et al., 2022), ICA-DP method demonstrates superior denoising while preserving the signals of interest.

In this paper, we introduce the use of ICA-DP to harmonize fMRI data collected from multiple sites. We apply ICA-DP to the data from Autism Brain Imaging Data Exchange II (ABIDE II) and compare its performance with two other common harmonization methods: ICA and ComBat. To assess the effectiveness of the harmonization methods, we utilize various techniques for visualizing and quantifying site effects before and after denoising. Additionally, we evaluate the denoising methods in terms of their ability to preserve signal effects.

## 2. Methods

### 2.1. Multi-site fMRI data

We utilized data from Autism Brain Imaging Data Exchange II (ABIDE II) to investigate the impact of site effects on ALFF and ReHo, and the performance of ICA-DP for denoising site effects and preserving signal effects.

Neuroimaging data from 1,114 subjects collected by 18 different sites with various scanner manufacturers (Simens, Philips, and GE) (Di Martino et al., 2017) were obtained from the ABIDE II dataset.^1^ We excluded images with obvious artifacts, large head movement (larger than one voxel size), and incomplete scanning of the whole brain. After strict quality control, functional MRI data of 795 subjects [Autism Spectrum Disorder (ASD) patients: 341, Healthy Controls (HC): 454] in 16 sites (data from two centers were fully excluded) were included in our study. The acquisition parameters: scanner and imaging-related details, including repetition time (TR), echo time (TE), flip angle (FA), voxel size, and demographic information (ASD/HC, sex, and age), are summarized in Table 1.

**Table 1:** Scanning parameters and demographic information of the multi-site ABIDE II data.

Sites	Scanners	TR/TE (ms)	FA (degree)	Voxel Size	ASD/HC	Male/Female	Age
EMC	GE MR750	2000/30	85	3.6 × 3.6 × 4.0	14/13	22/5	8.39 ± 1.03
ETH	Philips Achieva	2000/25	90	3 × 3 × 3	7/22	29/0	23.36 ± 4.59
GU	Siemens TriTim	2000/30	90	3 × 3×3	27/41	46/22	10.89 ± 1.62
IU	Siemens TriTim	813/28	60	3.4 × 3.4 × 3.4	18/19	28/9	24.62 ± 7.59
KKI	Philips Achieva	2500/30	75	3 × 3 × 3	25/123	89/59	10.37 ± 1.27
KUL	PhilipsAchieva	2500/30	90	1.6 × 1.6 × 3.1	25/0	25/0	23.76 ± 5.10
OHSU	Siemens TriTim	475/30	60	3 × 3 × 3	33/51	52/32	11.00 ± 2.04
ONRC	Siemens Skyra	2500/30	90	3.8 × 3.8 × 3.8	15/26	30/11	23.24 ± 4.09
SU	GE SIGNA	2000/30	80	3.4 × 3.4 × 3.5	14/17	28/3	10.94 ± 1.14
UCLA	Siemens TriTim	3000/28	90	3 × 3 × 4	12/12	19/5	11.04 ± 2.46
USM	Siemens TriTim	2000/28	90	3.1 × 3.1 × 4	9/12	17/4	24.34 ± 7.49
BNI	Philips Ingenia	3000/25	80	3.8 × 3.8 × 4	29/28	57/0	38.86 ± 15.41
IP	Philips Achieva	2700/45	90	3.6 × 3.7 × 4	13/21	16/18	22.37 ± 10.97
NYU	Siemens Allegra	2000/15	90	3 × 3 × 4	61/28	81/8	9.24 ± 4.78
SDSU	GE MR750	2000/30	90	3.4 × 3.4 × 3.4	30/24	46/8	13.19 ± 3.06
TCD	Philips Achieva	2000/27	90	3 × 3 × 3.2	9/17	26/0	15.98 ± 3.23

The data were collected from 13 different sites: Erasmus University Medical Center (EMC), ETH Zürich (ETH), Georgetown University (GU), Indiana University (IU), Kennedy Krieger Institute (KKI), Katholieke Universiteit Leuven (KUL), Oregon Health and Science University (OHSU), Olin Neuropsychiatry Research Center (ONRC), Stanford University (SU), University of California Los Angeles (UCLA), University of Utah School of Medicine (USM), Barrow Neurological Institute (BNI), Institut Pasteur and Robert Debré Hospital (IP), NYU Langone Medical Center (NYU), San Diego State University (SDSU), Trinity Centre for Health Sciences (TCD).

In this study, the site differences are defined as noise variables, and group differences (ASD/HC), age, and sex are regarded as signal variables. The correlation coefficients among these variables are summarized in Table 2. Since site differences are categorical variables, it is not achievable to directly calculate the correlation coefficients between categorical and numeric variables. We used ANOVA to calculate the significant levels of signal variables and site variables.

**Table 2:** The relationship of signal and noise variables.

Correlation	Site	ASD vs. HC	Age	Sex
Site	1.000(0.0000)	3.26e-18	2.29e-183	3.38e-18
ASD vs. HC	3.26e-18	1.000(0.0000)	**–**	0.1984(1.69e-8)
Age	2.29e-183	**–**	1.000 (0.0000)	−0.1223(5.50e-4)
Sex	3.38e-18	0.1984(1.69e-8)	−0.1223(5.50e-4)	1.000(0.0000)

### 2.2. Data preprocessing

The raw fMRI data were preprocessed with FSL FEAT, including removing the first six volumes, motion correction, and spatial normalization to standard MNI space. Two functional modalities, ALFF and ReHo, were generated from the preprocessed fMRI data with DPABI (Yan et al., 2016). For ReHo, spatial smoothing (with Full Width at Half Maximum (FWHM) of 6 mm) was performed after ReHo calculation, but for ALFF, spatial smoothing was completed before the calculation (Jia et al., 2019).

### 2.3. Harmonization methods

Two most widely used harmonization methods: ComBat and traditional ICA, were applied in this study to show the performance of ICA-DP on site-effects removal. We now describe the three different strategies below.

#### 2.3.1. ComBat

ComBat is a GLM-derived method based on empirical Bayes approach. The method assumes that the data can be modeled as a linear combination of signal variables and site effects, which includes additive and multiplicative factors:


(1) $$Y_{Non-denoised}=\alpha +X_{signal}\beta_{signal}+\gamma +\delta \varepsilon \;$$

where α is the average value, $X_{signal}$ is the design matrix for the signal variables and $\beta_{signal}$ is the corresponding regression coefficient, γ and δ are the additive and multiplicative factors, respectively. Then ComBat normalizes the data by removing the effects of average and signal variables:


(2) $$Y_{Normalized}=Y_{Non-denoised}-\alpha -X_{signal}\beta_{signal}\;$$

Finally, ComBat uses an empirical Bayes (EB) framework to get an improved estimate of site effects $\gamma^{*}$ and $\delta^{*}$ , after removing these site effects and adding the effects of average and signal variables back, we finally get the denoised data by ComBat:


(3) $$\begin{array}{c} Y_{Denoised}^{ComBat}=\frac{Y_{Non-denoised}-\alpha -X_{signal}\beta_{signal}-\gamma^{*}}{\delta^{*}}\\ +\alpha +X_{signal}\beta_{signal}\; \end{array}$$

#### 2.3.2. ICA and ICA-DP

ICA is a data-driven strategy that decomposes the data matrix into a set of statistically independent non-Gaussian maps together with associated courses (e.g., time, subject).


(4) $$Y_{Non-denoised}=A*S\;$$

where S is the spatial map and A is the corresponding courses. Compared with our ICA-DP, we rename the traditional ICA as ICA-SP (single-projection). To preserve the signal effects, ICA-SP method only removes those pure site-related components (related to site effects only), and leaves those mixed components without any process.


(5) $$Y_{Denoised}^{ICA-SP}=Y_{Non-denoised}-A_{Sites}pinv\left(A_{Sites}\right)Y_{Non-denoised}\;$$

where $A_{Sites}$ is the course of pure site-related components.

In order to eradicate the site effects, we proposed the ICA-DP method in our previous study (Hao et al., 2023). Firstly, ICA-DP separates the signal effects from the mixed components:


(6) $$A_{Sites}^{\prime }=A_{Mixed}-Var_{Signal}pinv\left(Var_{Signal}\right)A_{Mixed}\;$$

where $A_{Mixed}$ is the course of mixed components and $Var_{Signal}$ is the signal variable. Then $\left[\;A_{Sites}\;A_{Sites}^{\prime }\;\right]$ is utilized as the whole site effects to be regressed out.


(7) $$\begin{array}{c} Y_{Denoised}^{ICA-DP}=Y_{Non-denoised}-\left[\;A_{Sites}\;A_{Sites}^{\prime }\;\right]\\ pinv\left(\left[\;A_{Sites}\;A_{Sites}^{\prime }\;\right]\right)Y_{Non-denoised}\; \end{array}$$

### 2.4. Denoising process

For the ComBat-based method, the input $\;X_{signal}$ was set as group difference (ASD/HC), age, and sex.

For ICA-based methods, the data were decomposed into 100, 150, and 200 independent components. Pearson correlation and Analysis of Variance (ANOVA) were applied to identify signal, noise, and mixed components based on Hao et al. (2023). Components that only significantly correlated with the signal variables (*p* < 0.05, with Bonferroni correction) were classified as pure signal components. Conversely, those solely correlated with the noise variable were identified as pure noise components. Components related to both signal and noise variables were categorized as mixed components. For ALFF, we identified 79, 120, and 166 pure site-related components, and 21, 29, and 34 mixed components; For ReHo, we identified 83, 136, and 179 pure site-related components, and 15, 10, and 11 mixed components. The ICA-SP method exclusively utilized pure noise components to eliminate site effects, whereas the ICA-DP method employed all noise-related components, including mixed ones, for site effects removal. In the ICA-DP approach, both mixed and pure noise components were used to extract noise effects that are considered as integral site-related noise effects and used for removal. Both ICA methods were implemented using widely used software packages for neuroimaging data analysis, namely MATLAB and FSL MELODIC. FSLeyes^2^ and BrainNet (Xia et al., 2013) were used to present the results.

### 2.5. Evaluation of data denoising

We used several strategies to assess the performance of the three different denoising methods in terms of eliminating the site effects and preserving the signal effects. To visualize the site effects, we used t-distributed stochastic neighbor embedding (t-SNE) to observe the distribution of the data points, with a tendency to be clustered by site or not. Group F-test was also used to find the significant differences in ALFF and ReHo for brain regions associated with site differences. It is also important to show whether the methods can preserve the signal effects well. In this study, we used age, sex, and group difference (ASD/HC) as variables of interest. In addition to t-SNE and group-level tests of ASD vs. HC, the Pearson correlation coefficient between median ALFF, ReHo and age was also assessed. For each modality, the median value for each subject was obtained by calculating the median of 100 regions of interest.^3^ Then, the obtained values were sorted by age distribution, where different colors represent data from different sites.

## 3. Results

### 3.1. Visualization and quantification of site effects

**Figure 1:**
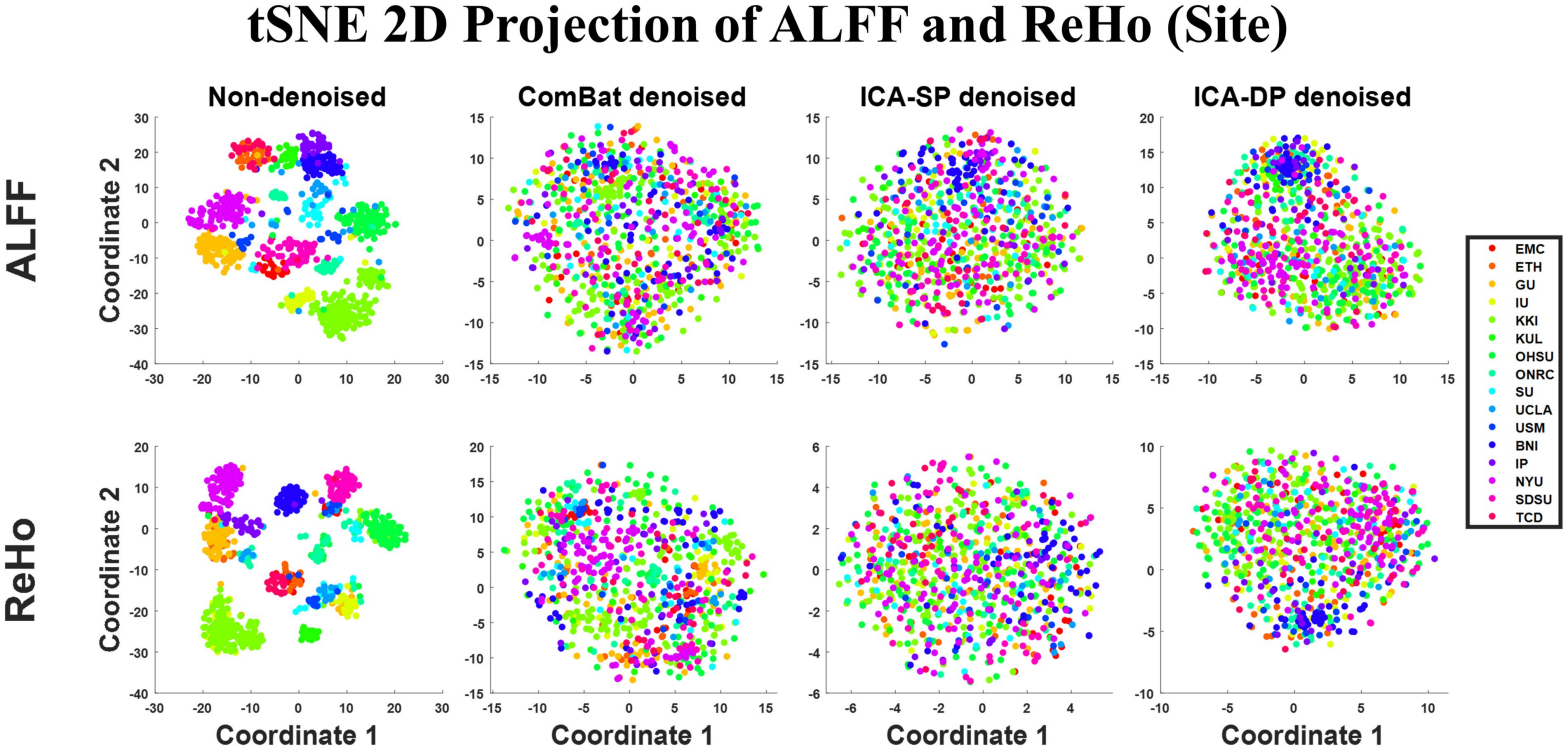
Dimension reduction visualization by t-SNE before and after denoising (Sites). The site-clustered distribution before denoising indicated the site effects, and it decreased when the data points were randomly distributed after denoising.

Figure 1 shows the tSNE-2D projection of ALFF and ReHo before and after site effects denoising. The tSNE can project the data into two vectors, which can be regarded as the two dominant features of the data. The data points of the non-denoised data showed site-clustered distribution as most of the centers had their own specific cluster areas, while this site-clustered distribution disappeared after being denoised by any of the denoising methods.

**Figure 2:**
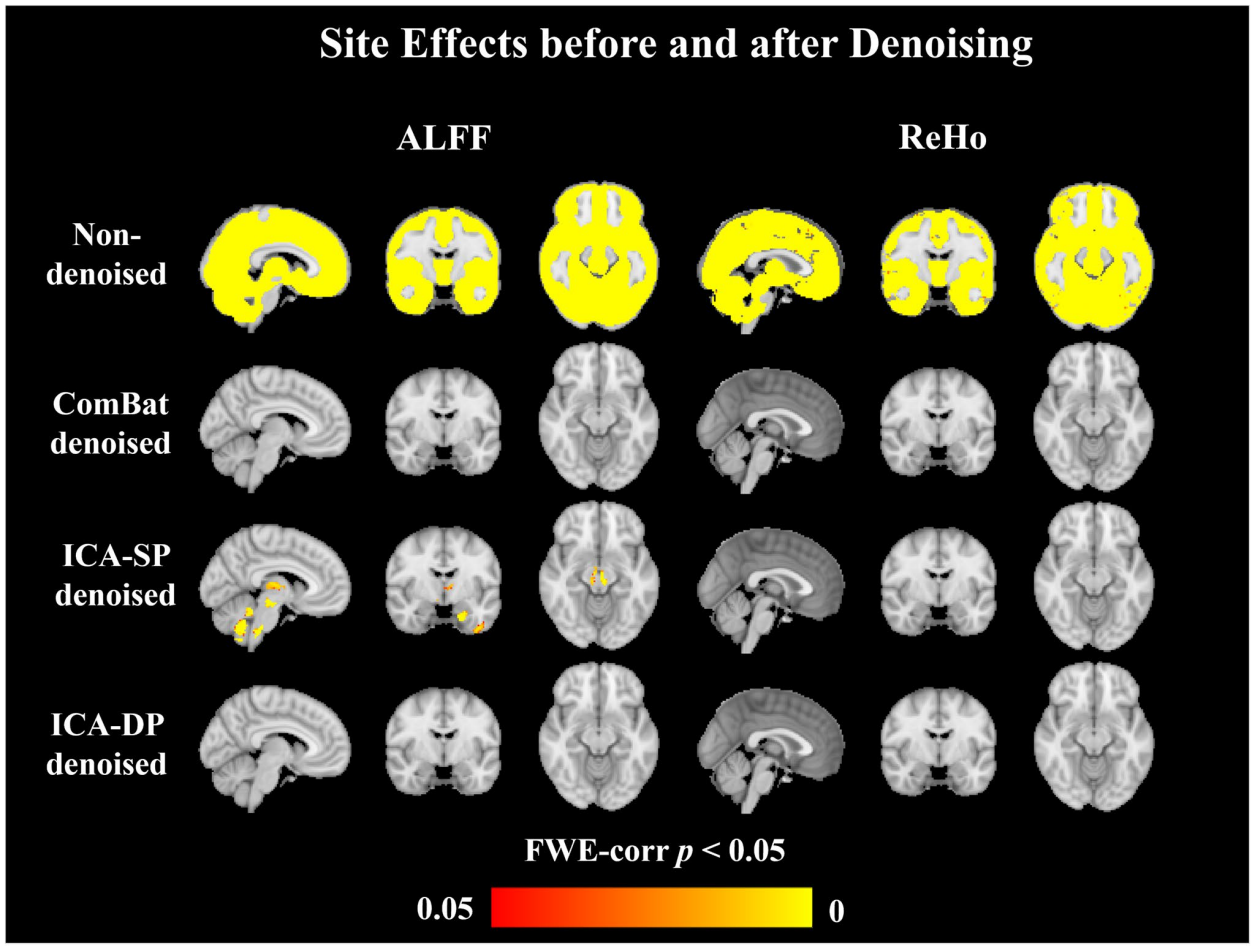
Group-level analysis for site effects before and after denoising. Site effects were removed entirely by ComBat and ICA-DP. Though ICA-SP reduced the site effects, some significant regions still could be found.

Figure 2 shows the group-level analysis for site effects. The analysis was based on a generalized linear model implementation of one-way ANOVA (factor: sites; covariates: age, sex, and group difference (ASD/HC)). Both two non-denoised modalities were globally contaminated by site effects. Though ICA-SP method decreased site effects, it was not very effective at denoising them. However, compared with our previous results applying ICA-SP to denoise site effects from structural MRI data (Hao et al., 2023), ICA-SP method did denoise site effects better for fMRI data, since purer site-related components were identified. After denoising with ICA-DP and ComBat, there were no brain regions with ALFF or ReHo that were associated with site difference (FWE-corrected *p* < 0.05).

### 3.2. Visualization and quantification of signal effects

#### 3.2.1. Age effects

**Figure 3:**
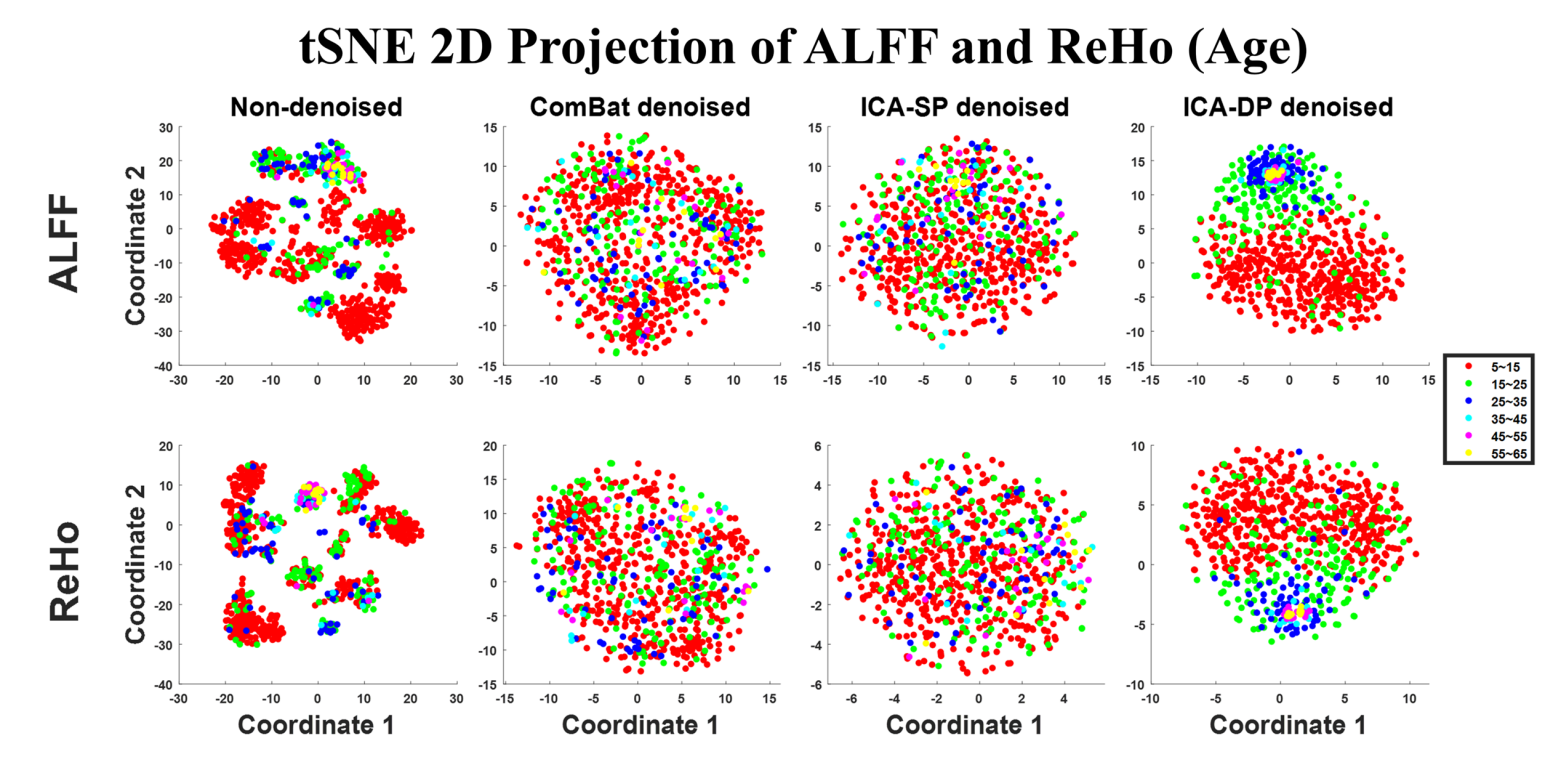
Dimension reduction visualization by t-SNE before and after denoising (Age). No age-clustered distribution before denoising, and the age-clustered distribution appeared after denoising by ICA-DP.

Figure 3 shows the tSNE-2D projection of ALFF and ReHo before and after site effects denoising. The data points of the non-denoised data did not show age-clustered distribution, while this age-clustered distribution appeared after being denoised by ICA-DP.

**Figure 4:**
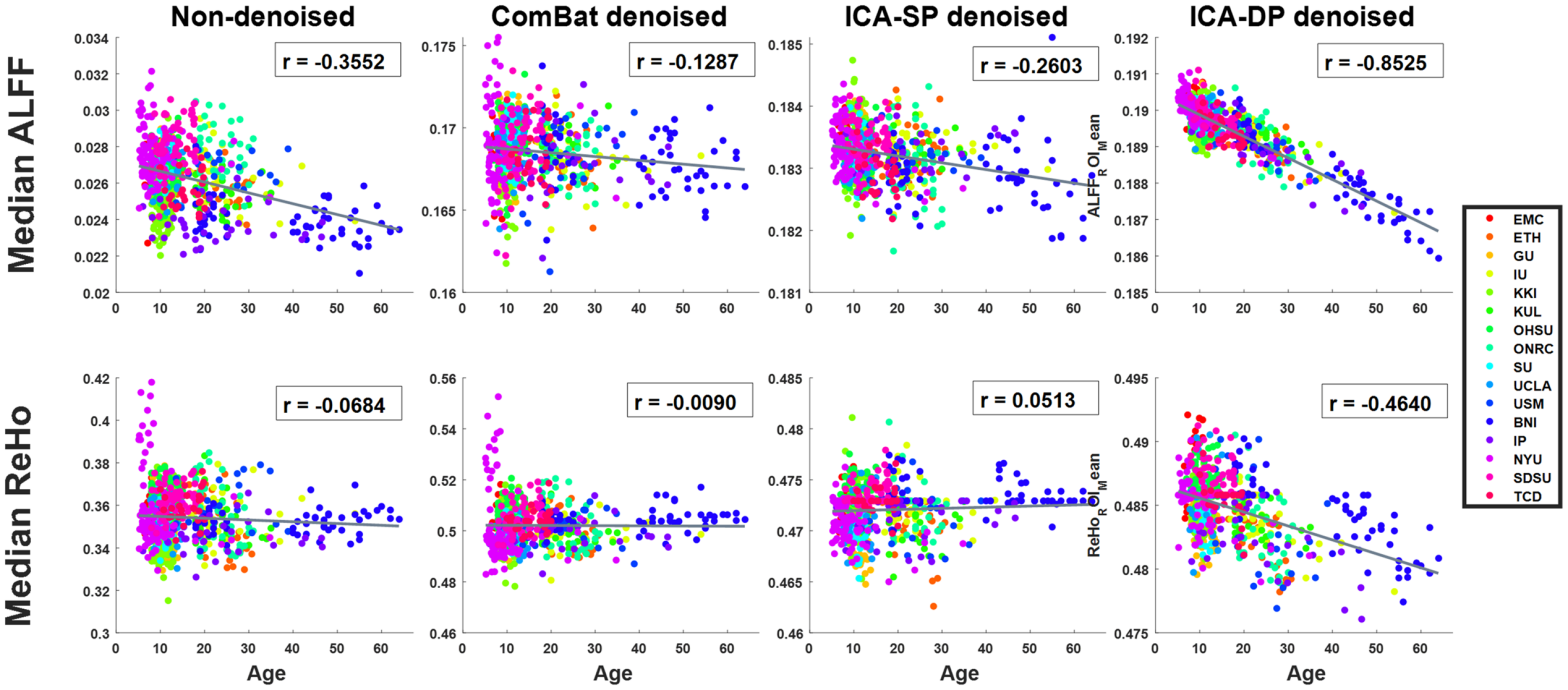
Relationship between age and whole-brain median ALFF, and ReHo. For ALFF, the Pearson correlation coefficients were − 0.3552 (Non-denoised), −0.1287 (ComBat denoised), −0.2603 (ICA-SP denoised), −0.8525 (ICA-DP denoised), respectively; for ReHo, the Pearson correlation coefficients were − 0.0684 (Non-denoised), −0.0090 (ComBat denoised), 0.0513 (ICA-SP denoised), −0.4640 (ICA-DP denoised), respectively.

Figure 4 displays the correlation between the median values of the connectivity measures across the whole brain and age for each of the two modalities. For ALFF, the Pearson correlation coefficients were − 0.3552 (Non-denoised), −0.1287 (ComBat denoised), −0.2603 (ICA-SP denoised), −0.8525 (ICA-DP denoised), respectively. For ReHo, the Pearson correlation coefficients were − 0.0684 (Non-denoised), −0.0090 (ComBat denoised), 0.0513 (ICA-SP denoised), −0.4640 (ICA-DP denoised), respectively. From a whole-brain perspective, only the ICA-DP method enhanced the correlation between the two modalities and age.

**Figure 5:**
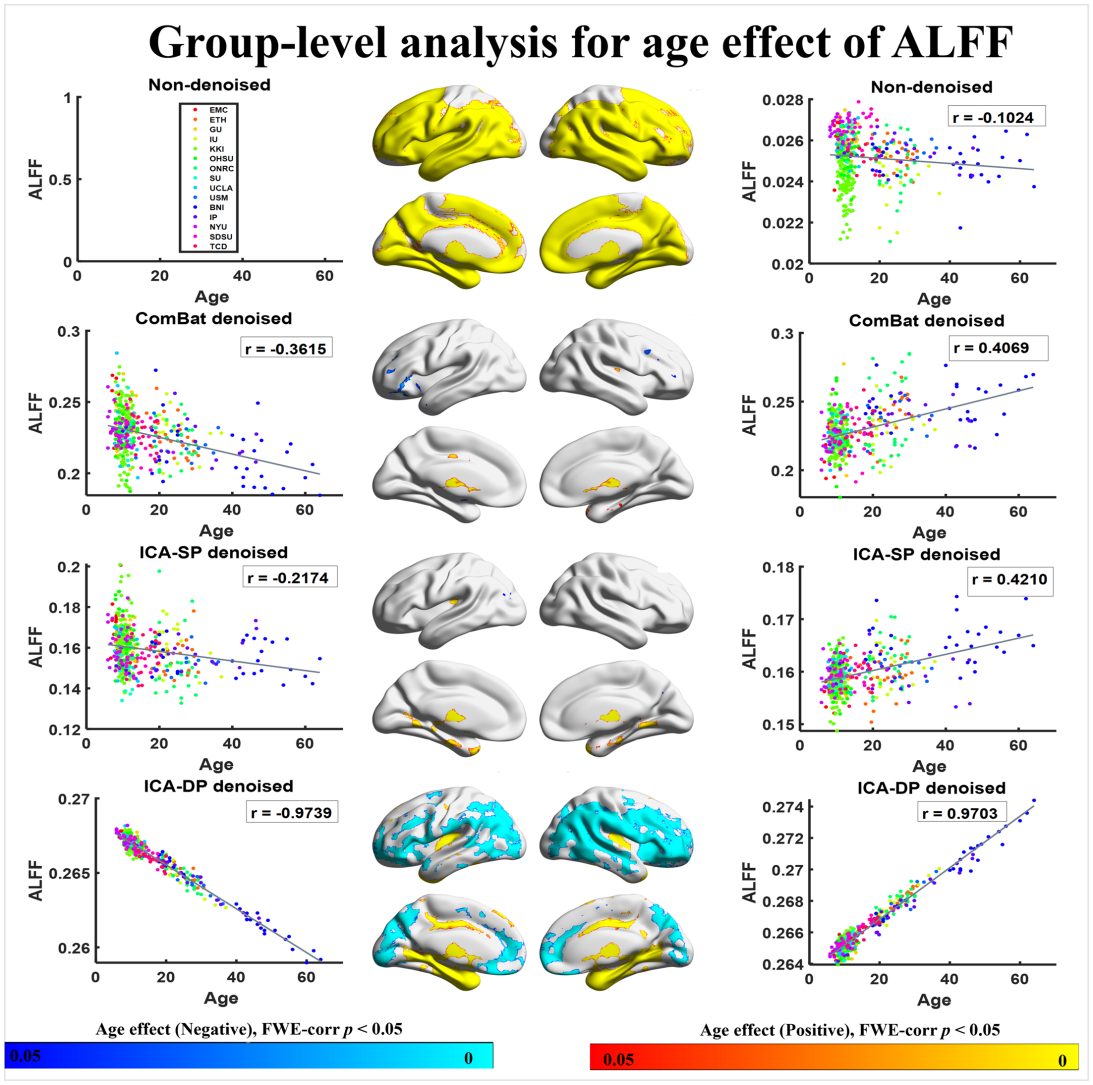
Associations between age and ALFF with different denoising strategies. “Positive” association indicates increasing amplitude with increasing age, whereas “Negative” refers to decreasing amplitude with increasing age. Associations with age are enhanced by ICA-DP and weakened by ICA-SP and ComBat.

**Figure 6:**
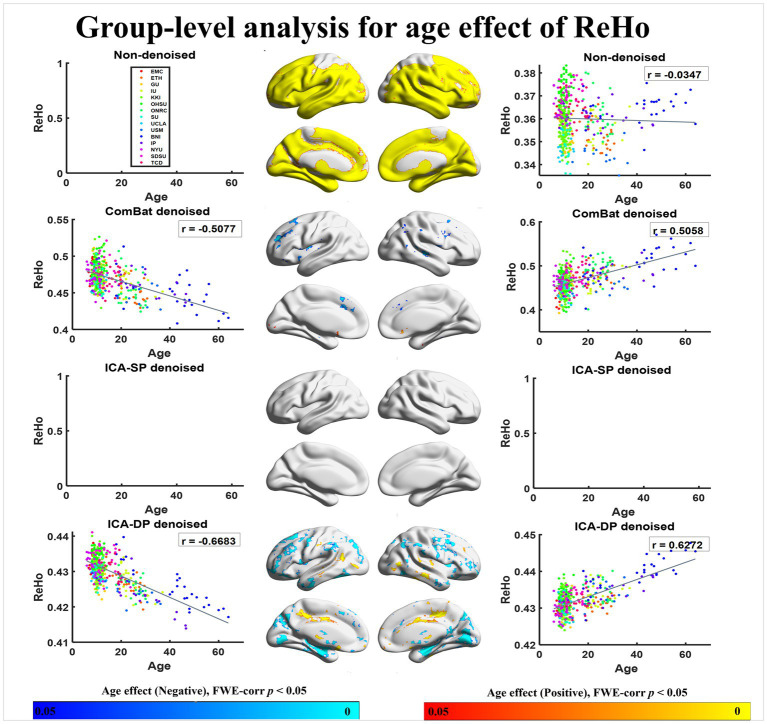
Associations between age and ReHo with different denoising strategies. “Positive” refers to significantly increasing amplitude with increasing age, whereas “Negative” refers to significantly increasing amplitude with decreasing age. The age effects are enhanced by ICA-DP, while weakened by ICA-SP and ComBat.

Figures 5, 6 show the group-level analyses for age on ALFF and ReHo, and correlations between age-related regions and age. In order to better rule out the influence of ASD, we only analyzed the age effect of healthy people. The group-level analyses were based on a generalized linear model implementation of one-way ANOVA (factor: age; covariates: sex). Figure 5 shows the results from ALFF. The negative age effects were not found in the non-denoised data because of the existence of site effects, removal of the effects by all the denoising methods, especially for ICA-DP, could reveal the negative age effects not detected from the non-denoised data. From the results of ICA-DP, regions positively associated with age included Cerebellum, Thalamus, Temporal Lobe, and Frontal Lobe; regions negatively associated with age included Parietal Lobe, Temporal Lobe, and Frontal Lobe for the non-denoised data. Figure 6 shows the results from ReHo. The results had the same tendency as those from ALFF. Site effects masked the negative age effects. Removal of the effects by ComBat and ICA-DP could reveal the negative age effects not detected from the non-denoised data. There were no age effects detected after denoising by ICA-SP. From the results of ICA-DP, regions positively associated with age included the Frontal Lobe, Parietal Lobe, and Temporal Lobe; regions negatively associated with age included Occipital Lobe, and Parietal Lobe.

In summary, ICA-DP increased the age effects by detecting more significantly different regions related to age, while ComBat and ICA-SP decreased the age effects with fewer or no significant regions.

#### 3.2.2. Sex effects

**Figure 7:**
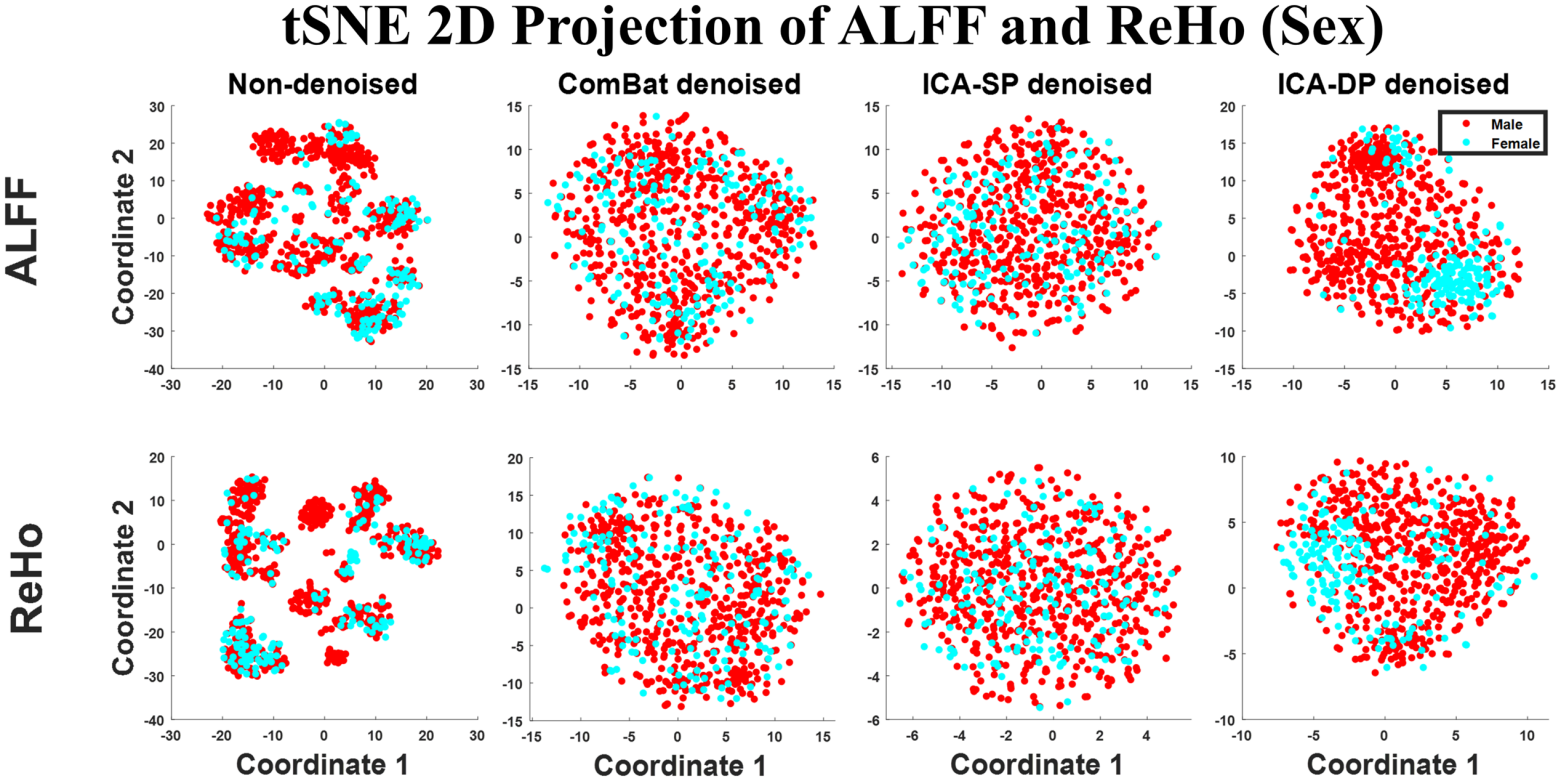
Dimension reduction visualization by t-SNE before and after denoising (Sex). No sex-clustered distribution before denoising, and there was a light sex-clustered tendency after denoising by ICA-DP.

Figure 7 shows the tSNE-2D projection of ALFF and ReHo before and after site effects denoising. The data points of the non-denoised data did not show sex-clustered distribution, which appeared after being denoised by ICA-DP.

**Figure 8:**
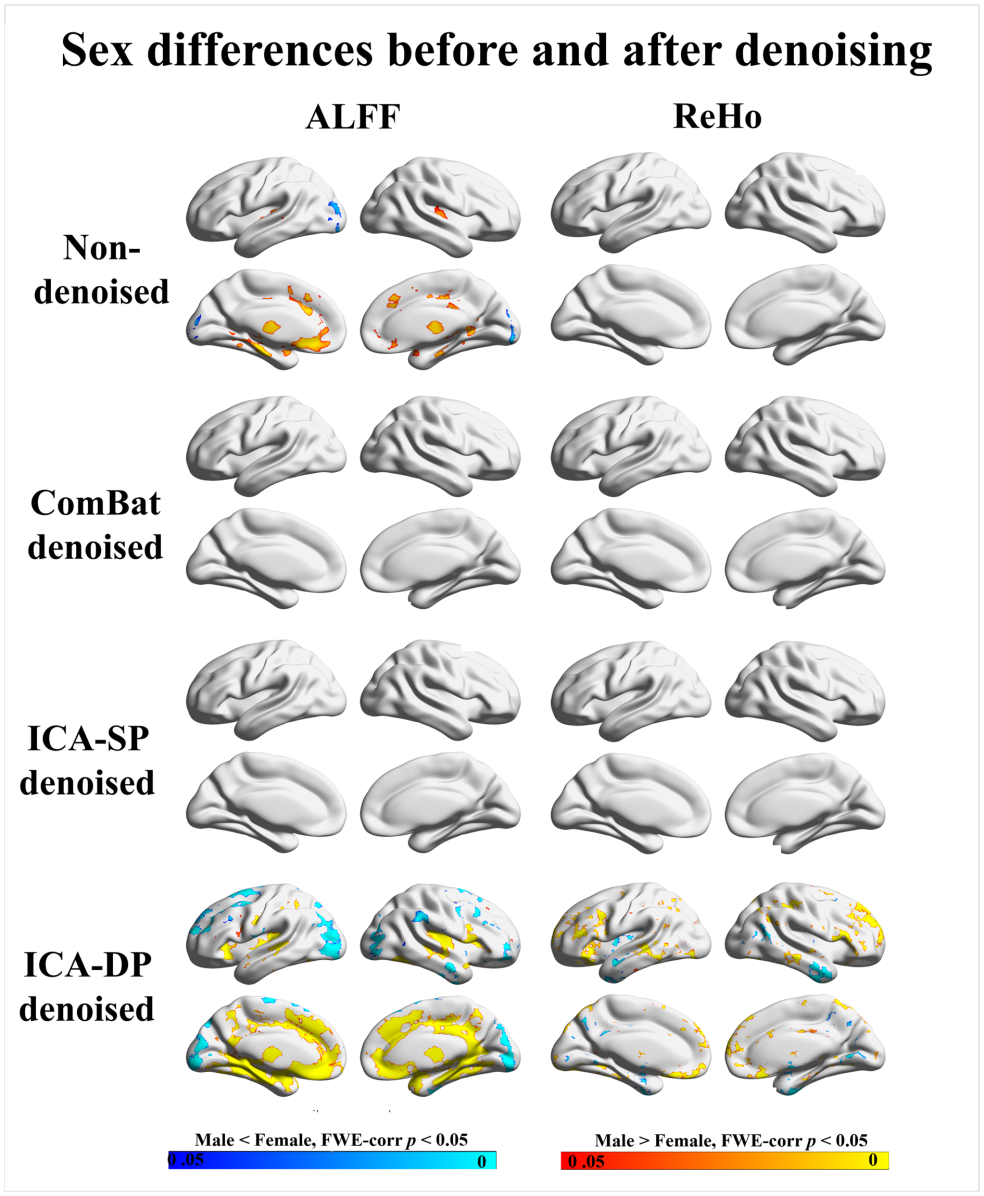
Sex differences before and after denoising. “Male < Female” refers to significantly greater amplitude in females, whereas “Male > Female” refers to significantly greater amplitude in males. The sex effects are enhanced by ICA-DP, while weakened by ICA-SP and ComBat.

Figure 8 displays the group-level analyses for sex on the two fMRI modalities. The group-level analyses were based on a generalized linear model implementation of one-way ANOVA (factor: sex; covariates: age, and group difference (ASD/HC)). For ALFF, we observed several regions that were significantly greater in males, including the Frontal Lobe, Thalamus, and Temporal Lobe.; regions significantly greater in females included Occipital Lobe for the non-denoised data. After denoising, our ICA-DP widened the boundaries of these regions, while the other two methods resulted in the disappearance of these regions. For ReHo, no regions were associated with sex. After denoising with ICA-DP, we identified regions associated with sex. Specifically, regions significantly greater in males included Frontal Lobe, Parietal Lobe, and Occipital Lobe; regions significantly greater in females included Cerebellum, and Temporal Lobe.

Similar to the results for age effects, ICA-DP increased the sex effects by detecting more significantly different regions related to sex, while ComBat and ICA-SP decreased the sex effects with fewer or less significant regions.

#### 3.2.3. Group difference (ASD/HC)

**Figure 9:**
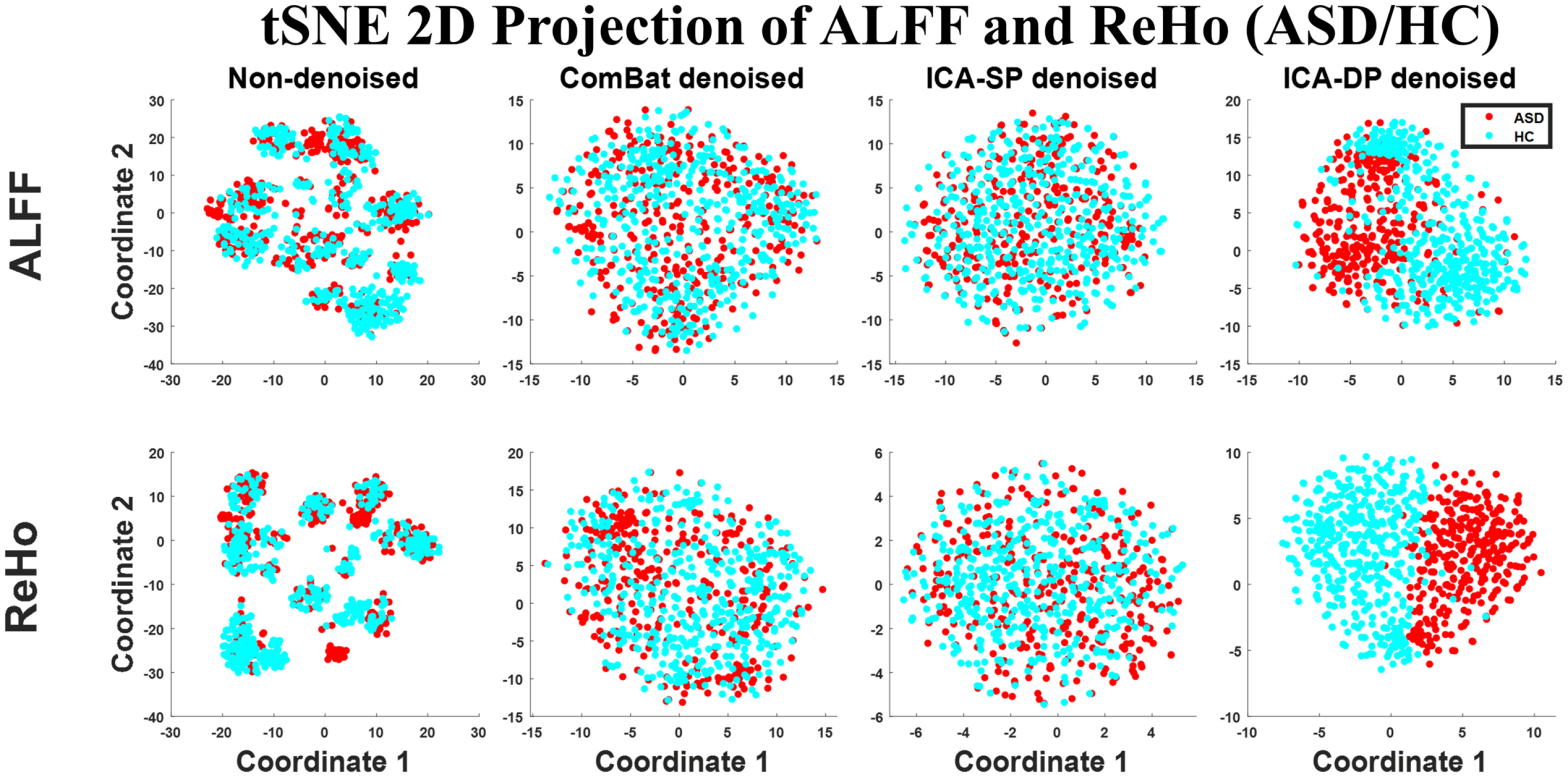
Dimension reduction visualization by t-SNE before and after denoising (ASD vs. HC). The data points of the non-denoised data were randomly distributed, and only the data points after denoising by ICA-DP could be divided into two groups according to the group difference (ASD/HC).

Figure 9 shows the tSNE-2D projection of ALFF and ReHo before and after site effects denoising. The data points of the non-denoised data could not be divided into two groups according to the group difference (ASD/HC), and only ICA-DP method could enhance the group effects by distinguishing ASD and HC.

**Figure 10:**
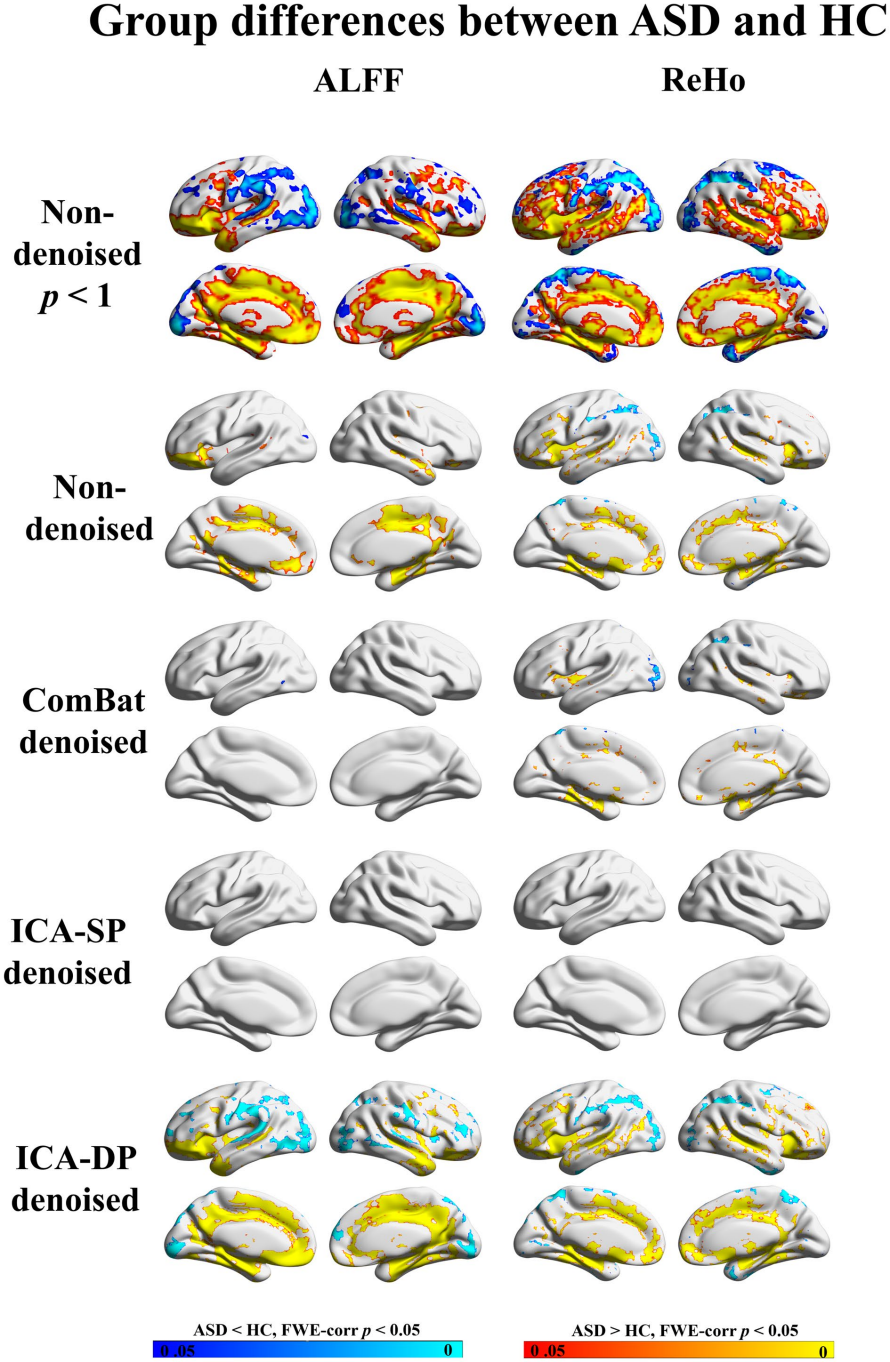
Group differences between ASD and HC before and after denoising. No significant differences in regions were found from the data denoised by ICA-SP, and fewer regions were found from the data denoised by ComBat, while ICA-DP could increase the significance of the regions related to ASD/HC. FWE-corr *p* < 1 was shown for non-denoised data to indicate that the regions tested from ICA-DP denoised data were not reintroduced artifacts.

Figure 10 demonstrates the impact of denoising on group differences between individuals with autism spectrum disorder (ASD) and healthy controls (HC). The group-level analyses were based on a generalized linear model implementation of one-way ANOVA (factor: group difference (ASD/HC); covariates: age and sex). The results revealed that ICA-DP enhanced the group effects by identifying more regions that were significantly different between the two groups, whereas ComBat and ICA-SP decreased the group effects by detecting fewer or less significant regions. When compared to the non-thresholded group difference maps from the original data (first row), it could be seen that the regions associated with group differences (ASD/HC) from ICA-DP-based denoised data were also present in the original data. This suggested that ICA-DP only amplified the existing signal and did not introduce new information.

## 4. Discussion

In this study, we applied the ICA-DP method to the multi-site harmonization of ALFF and ReHo, and compared it to traditional ICA and ComBat methods for removing site effects and preserving biological variability. The results showed that our ICA-DP method can better remove site effects and preserve physiological signals compared with two other approaches for denoising, ICA-SP, and ComBat.

In the non-denoised data, site effects objectively exist in both modalities: 1) original ALFF and ReHo both show a trend of clustering by site (Figure 1), even if the data from the same site have different distributions of age, sex, and group difference (ASD/HC). To some extent, the statistical differences caused by site differences are greater than those caused by other biological variables (Figures 3, 7, 9). Moreover, these large site differences may mask the examination of biological effects (Eshaghzadeh Torbati et al., 2021). 2) The results of the F-test indicate that the two modalities all show obvious site differences, and the impact is whole-brain.

Regarding denoising performance, both ICA-DP and ComBat methods can thoroughly remove site effects: (1) the denoised data no longer clusters by site; F-test results no longer have significantly correlated activation regions with site variables. The traditional ICA can only remove the effects of site effects to some extent and cannot eradicate it, because the traditional ICA method only removes the influence of pure noise components and does not deal with mixed components. If the proportion of mixed components in all components is relatively large, or the site effects in mixed components are relatively apparent, the denoising ability of the traditional ICA method will be greatly discounted; on the contrary, the ICA method will have better denoising effect.

In addition to evaluating the performance of the three methods in removing site effects, it is equally important to evaluate their ability to preserve biological signals. To this end, we define age, sex, and group difference (ASD/HC) as variables of interest. The results show that our ICA-DP method effectively removes site effects while also enhancing the examination of biological signals, including the effects of age, sex, and group difference (ASD/HC). The other two methods reduced the examination of these biological effects. Our method’s enhancement of biological signals is due to the fact that for each noise component identified, we first regress out the influence of biological signals and then use it for denoising so that the proportion of physiological signals in the denoised data is relatively large and it is easier to detect brain regions that are related to signals through statistical tests. From another perspective, this might result in other variables, in which we are not interested, not being well preserved (Hao et al., 2023). The other limitation of the proposed harmonization method is that when the noise variable is strongly related to the signal variable, ICA-DP could not eliminate the intersection effects related to both site and signal variables.

In 2010, Biswal et al. conducted a study on age and sex in a large sample of fMRI data from 35 sites. They also reported site effects. Although they did not remove the site effect in their study and just utilized sites as covariates in a generalized linear model (GLM), they still identified some brain regions that were significantly correlated with age and sex in the ALFF. In some of our results (age and sex effect of ALFF), we also found activation regions that highly overlap with Biswal’s results. Because we used different datasets and different sample sizes, our results are highly overlapping, but not exactly the same. In addition, we believe that if we apply our method to their dataset and remove the site effect with ICA-DP, more similar activation regions related to age and sex will be founded.

Regarding the statistical results of group differences between individuals with autism spectrum disorder (ASD) and healthy controls (HC), our research identified similar brain regions that have been highlighted in previous studies. For example, in patients with ASD, increased ALFF in the Temporal Lobe and Frontal Lobe while decreased ALFF in the Occipital Lobe was also found compared to HC (Wang et al., 2023). Participants with ASD also showed increased ReHo in the Frontal Lobe and decreased ReHo in the Temporal and Frontal Lobe compared to HC (Paakki et al., 2010; Canario et al., 2021; Wang et al., 2023).

To the best of our knowledge, there are only a few studies focused on the harmonization of multiple sites for ALFF and ReHo to reveal their associations with age, sex, and group difference (ASD/HC). Thus, we are cautious in interpreting the results until the same results can be repeated on a large sample dataset from a single center.

## 5. Conclusion

The combination of multi-site MRI data has the potential to increase the statistical power and improve the reliability and reproducibility of neuroimaging research. However, the analysis of MRI data is often confounded by site effects. Removing these site effects is a critical step in the process of multi-site data fusion. In addition, preserving signals of interest is a major concern when applying any denoising strategy. ICA-SP and ComBat reduced associations with age and sex.

In contrast, our ICA-DP method has proven to be effective in removing site effects and preserving biological variability. With our ICA-DP method, multi-site fMRI data can be harmonized, thus allowing for more robust and accurate analysis. This approach can significantly enhance the validity of neuroimaging research, and we believe it will be a valuable tool for future studies.

## Data availability statement

Publicly available datasets were analyzed in this study. This data can be found at: http://fcon_1000.projects.nitrc.org/indi/abide/abide_II.html.

## Author contributions

HX wrote the manuscript with comments from TK, HL, LN, and FC. YZ and DZ downloaded the data and preprocessed them. YH contributed to the guidance of methods. HX carried out the data analysis. All authors read and approved the final manuscript.

## Funding

This work was supported by STI 2030–Major Projects 2022ZD0211500, Science and Technology Planning Project of Liaoning Provincial (nos. 2022JH2/10700002 and 2021JH1/10400049), National Natural Science Foundation of China [grant numbers 91748105 and 81471742], National Foundation in China [grant number JCKY 2019110B009], and National Institutes of Health [NIA RF1 AG078304].

## Conflict of interest

The authors declare that the research was conducted in the absence of any commercial or financial relationships that could be construed as a potential conflict of interest.

## Publisher’s note

All claims expressed in this article are solely those of the authors and do not necessarily represent those of their affiliated organizations, or those of the publisher, the editors and the reviewers. Any product that may be evaluated in this article, or claim that may be made by its manufacturer, is not guaranteed or endorsed by the publisher.

## Footnotes

^1^http://fcon_1000.projects.nitrc.org/indi/abide/ abide_II.html

^2^https://fsl.fmrib.ox.ac.uk/fsl/fslwiki/FSLeyes

^3^https://github.com/ThomasYeoLab/CBIG/tree/master /stable_projects/brain_ parcellation/Schaefer2018_LocalGlobal/Parcellations/
